# Characteristics of respirable dust in eight appalachian coal mines: A dataset including particle size and mineralogy distributions, and metal and trace element mass concentrations

**DOI:** 10.1016/j.dib.2019.104032

**Published:** 2019-05-22

**Authors:** Emily Sarver, Cigdem Keles, Mohammad Rezaee

**Affiliations:** a Department of Mining and Minerals Engineering, Virginia Tech, Blacksburg, VA 24061, USA; b John and Willie Leone Family Department of Energy and Mineral Engineering, Earth and Mineral Sciences (EMS) Energy Institute, The Pennsylvania State University, University Park, PA 16802, USA

**Keywords:** Respirable dust, Coal mining, Occupational health, Black lung, Mining engineering

## Abstract

Respirable dust samples were collected in several key locations of eight underground coal mines in central and northern Appalachia. In total, there were 76 unique sampling events (i.e., specific location in a specific mine). Here, we present data from each event describing particle size and mineralogy class distributions across the ∼100–10,000nm size range, which were determined using SEM-EDX; and estimated mass concentrations of potentially bioaccessible and total acid-soluble metals and trace elements, which were determined using sequential digestions with digestate analysis by ICP-MS. Discussion of this dataset is included in a companion research article “Beyond conventional metrics: Comprehensive characterization of respirable coal mine dust” Sarver et al., 2019.

**Table:** Specifications table

Subject area	Engineering
More specific subject area	Environmental monitoring for occupational health, mining engineering
Type of data	Table (i.e., summary of 50 + variables); graphs (i.e., particle size distributions for each mineralogy class by mine, sampling location); descriptive information on methods including necessary tables/figures is also provided (e.g., to describe digestion solutions).
How data was acquired	Particle size and mineralogy distribution: this was done by SEM-EDX using an FEI Quanta 600 FEG environmental SEM (FEI, Hillsboro, OR) equipped with a Bruker Quantax 400 EDX spectroscope (Bruker, Ewing, NJ) Potentially bioaccessible and total acid-soluble metal and trace element concentrations: the digestate solutions were analyzed by ICP-MS using a Thermo Electron X Series instrument (Thermo Fisher Scientific, Waltham, MA).
Data format	Raw and analyzed
Experimental factors	Respirable samples were collected onto polycarbonate filters. SEM-EDX work was performed directly on the filter media after sputter-coating with Au/Pd. For the metals and trace elements analysis, dust was removed from the filters by sonication, and then digested in simulated lung fluid and then strong acid.
Experimental features	Particle size and mineralogy distribution: Data in the supramicron ranges was collected using a computer-controlled SEM-EDX routine, which we have already described in detail elsewhere (see [5]). Data in the submicron range was collected by manual SEM-EDX, and the method is described in detail here and summarized in the companion article. Data was merged across the two size ranges by normalizing particle counts on a unit of analyzed-filter-area basis. Potentially bioaccessible and total acid-soluble metal and trace element estimated concentrations: the digestion to determine potentially bioaccessible elements used for this work was adapted from a published method [6], and that used for total acid-soluble elements is adapted from ASTM D7439-14[8]. We provide a detailed description of the entire method for our samples in the current article.
Data source location	Samples were collected in 3 distinct regions of Appalachia. We are under non-disclosure agreements with industry partners to keep actual mine identities anonymous, but have published the general locations and mine descriptions (see [2]).
Data accessibility	Within this article.
Related research article	Ref [1]: E. Sarver, C. Keles, M. Rezaee, Beyond conventional metrics: comprehensive characterization of respirable coal mine dust, Int. J. Coal Geol. 207 (2019) 84–95. https://doi.org/10.1016/j.coal.2019.03.015.

**Table:** 

**Value of the data** • This dataset represents a comprehensive characterization of respirable coal mine dust. • The data may inform a ranged of stakeholders interested in respirable dust, including those in industry such as mine operators and miners; those in the health sciences including epidemiologists, toxicologists and pathologists; and those in engineering and technology development for dust controls and protections. • The additional value of the data is that the specific dust characteristics included here have not been widely reported elsewhere in the literature.

## 1 Data

**Fig. 1:**
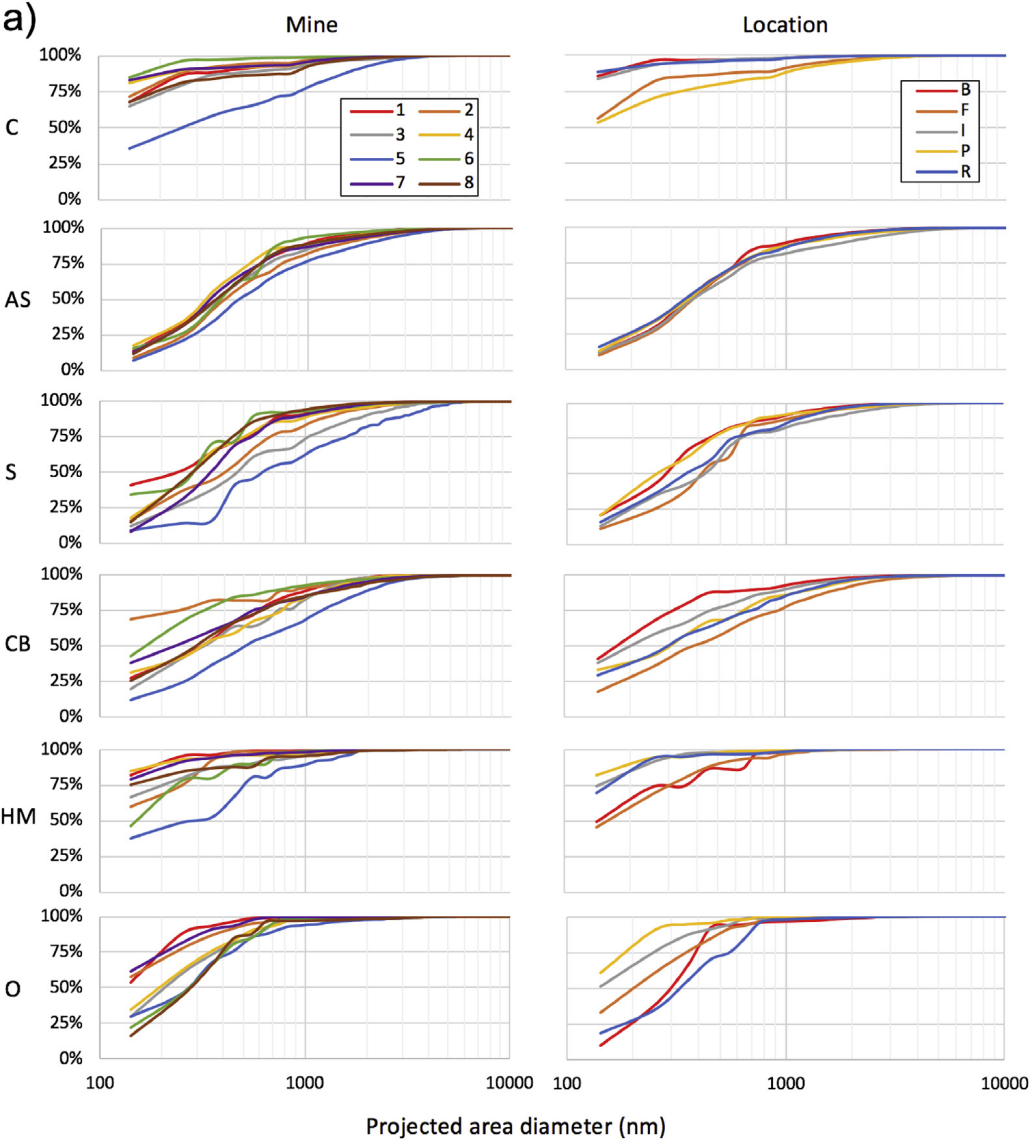

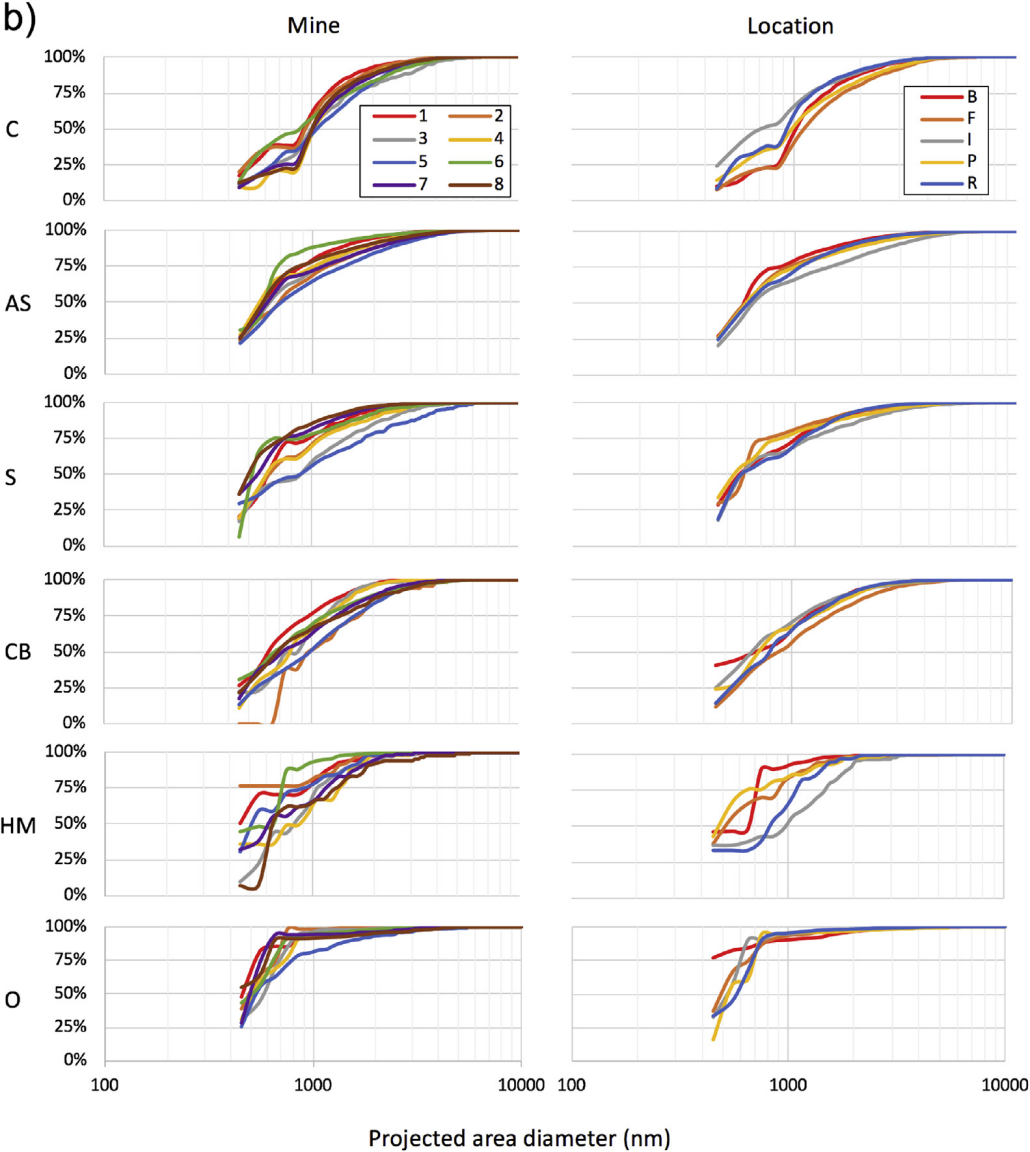
Cumulative particle size distributions for a) the entire analyzed size range and b) for >400nm particles. Data are shown for each mineralogy class and presented by mine (left) and sampling location (right). Since the number of samples from each location varied by mine, results were averaged for each location in each mine (i.e., n = 39). To generate these plots, 100-nm wide bin sizes were considered. Reproduced from the companion research article [1].

This dataset includes 76 respirable coal mine dust samples, which were collected in five general locations of eight underground coal mines in Appalachia. Each sample represents a unique sampling event (i.e., specific sampling location in a specific mine). Table 1 presents a summary of the particle size and mineralogy distribution results for each sample. For this summary, particles were binned into two primary size bins using their projected area diameter: very fine (i.e., <400nm) and larger particles (i.e., 400–10,000nm). Particles were binned into six mineralogy classes (i.e., carbonaceous, alumino-silicates, silica, carbonates and heavy minerals, or “other”). Fig. 1 presents more detailed size distribution data by mine and sampling location.

**Table 1:** Summary of particle characteristics for 76 respirable coal mine dust samples. Samples are ordered by mine region (i.e., MCA = mid-central Appalachia, NA = northern Appalachia, SCA = south-central Appalachia), mine number (i.e., 1–8), sampling location (i.e., I = intake, R = return, P = production, B = bolter, F = feeder). Mineralogy classes are C = carbonaceous, AS = alumino-silicates, S = silica, CB = carbonates, HM = heavy minerals, O = other.

Sample				% of particles in size and mineralogy class													
				C		AS		S		CB		HM		O		Total	
No.	Reg.	Mine	Loc.	Very fine	Larger	Very fine	Larger	Very fine	Larger	Very fine	Larger	Very fine	Larger	Very fine	Larger	Very fine	Larger
1	MCA	1	F	7	4	18	22	2	2	8	5	8	1	17	5	60	40
2	MCA	1	B	0	1	55	36	2	3	0	0	0	0	2	0	59	41
3	MCA	1	I	19	1	5	9	1	0	24	26	6	0	7	1	62	38
4	MCA	1	R	2	1	45	45	2	2	1	0	1	0	1	0	52	48
5	MCA	1	B	38	16	10	7	1	5	1	2	3	1	9	7	61	39
6	MCA	1	P	18	1	4	5	3	0	12	8	21	0	28	0	86	14
7	MCA	1	B	2	1	39	51	2	4	0	0	0	0	1	0	44	56
8	MCA	2	I	36	3	10	15	4	4	6	1	12	0	7	1	75	25
9	MCA	2	R	0	0	25	26	23	26	0	0	0	0	0	0	48	52
10	MCA	2	B	0	0	21	36	19	24	0	0	0	0	0	0	40	60
11	MCA	2	P	31	4	10	15	9	8	0	0	0	0	19	3	69	31
12	MCA	2	F	49	4	15	15	1	4	0	0	2	1	9	1	76	24
13	MCA	2	B	0	0	29	66	2	3	0	0	0	0	0	0	31	69
14	MCA	3	I	31	2	10	11	1	2	14	10	6	0	11	2	72	28
15	MCA	3	R	7	2	34	45	4	5	0	0	3	1	0	0	47	53
16	MCA	3	B	23	4	21	24	2	3	7	8	3	1	3	2	58	42
17	MCA	3	P	2	1	45	44	2	3	0	0	1	0	0	1	51	49
18	MCA	3	F	44	9	17	17	1	3	0	1	1	1	4	3	67	33
19	MCA	4	I	21	2	37	25	2	5	2	4	1	0	1	0	63	37
20	MCA	4	R	27	1	5	6	1	0	22	17	9	0	8	3	73	27
21	MCA	4	B	6	2	34	26	21	9	0	0	2	0	1	0	63	37
22	MCA	4	F	39	11	6	5	1	2	0	2	11	2	13	9	70	30
23	NA	5	R	5	5	28	54	1	2	1	2	1	0	1	0	36	64
24	NA	5	I	40	4	3	17	1	8	4	14	1	1	5	2	54	46
25	NA	5	I	17	15	4	25	1	2	7	23	2	1	3	1	34	66
26	NA	5	F	19	3	5	12	2	2	23	27	1	0	4	2	54	46
27	NA	5	P	10	10	29	37	0	1	3	4	1	1	3	1	45	55
28	NA	5	R	3	8	26	44	1	1	4	7	1	0	4	1	38	62
29	NA	5	B	8	14	9	27	1	3	12	22	1	1	1	1	31	69
30	NA	5	F	5	9	8	13	0	0	16	28	2	0	11	7	41	59
31	NA	5	F	7	10	4	14	0	2	17	37	1	0	3	2	33	67
32	NA	5	R	1	0	11	23	0	1	26	33	0	0	2	2	41	59
33	NA	6	I	21	2	17	15	0	0	21	14	6	1	2	1	66	34
34	NA	6	R	4	0	1	3	0	0	43	46	0	0	1	2	49	51
35	NA	6	F	17	2	26	23	1	1	12	6	5	1	4	2	65	35
36	NA	6	I	17	1	4	3	0	1	34	38	1	0	1	0	56	44
37	NA	6	R	1	0	1	4	0	0	19	72	0	0	1	0	23	77
38	NA	6	P	34	40	8	10	0	0	2	4	1	1	1	1	45	55
39	NA	6	I	21	6	2	3	1	0	20	44	1	0	2	1	46	54
40	NA	6	R	32	5	5	9	1	1	20	23	2	1	0	0	61	39
41	NA	6	F	63	4	6	4	2	1	3	8	3	1	2	1	80	20
42	NA	6	B	67	0	4	5	1	0	14	2	4	1	1	1	90	10
43	NA	6	I	74	1	1	1	1	0	7	6	2	0	6	1	90	10
44	NA	6	R	82	1	2	0	0	1	6	1	3	0	1	2	94	6
45	NA	6	I	38	1	0	5	0	0	12	5	10	1	26	1	86	14
46	NA	6	I	28	9	21	15	0	1	5	5	2	1	9	4	65	35
47	NA	6	F	37	2	1	0	1	0	51	5	0	0	2	1	92	8
48	SCA	7	R	16	5	39	21	4	3	6	1	2	1	2	0	69	31
49	SCA	7	I	56	2	7	6	0	2	0	4	0	0	22	2	85	15
50	SCA	7	R	5	8	44	32	2	2	4	2	1	0	0	0	56	44
51	SCA	7	I	66	2	2	2	1	1	8	2	5	0	10	1	93	7
52	SCA	7	F	4	13	15	30	1	2	10	19	1	0	2	2	34	66
53	SCA	7	P	12	11	20	24	1	1	15	4	1	0	9	1	58	42
54	SCA	7	P	7	8	36	24	6	3	3	3	3	0	7	1	61	39
55	SCA	7	F	2	11	29	45	5	4	0	1	1	0	1	1	38	62
56	SCA	7	B	2	9	33	27	4	3	7	12	1	1	1	1	48	52
57	SCA	7	R	1	4	37	36	8	9	1	2	1	0	0	0	48	52
58	SCA	7	P	3	7	40	29	3	2	6	9	0	0	1	0	54	46
59	SCA	7	B	4	7	37	42	4	3	0	1	0	0	1	0	47	53
60	SCA	7	F	1	5	39	45	2	3	1	3	0	0	0	0	43	57
61	SCA	7	P	2	5	41	40	2	4	1	3	0	0	0	0	47	53
62	SCA	8	R	0	0	17	34	0	0	21	26	0	0	1	1	39	61
63	SCA	8	P	12	3	34	25	18	7	0	0	0	0	0	0	64	36
64	SCA	8	I	29	5	14	31	2	5	4	7	2	0	1	0	52	48
65	SCA	8	F	5	10	21	51	2	3	3	4	0	0	1	0	32	68
66	SCA	8	I	59	2	13	10	1	1	4	0	3	0	5	3	84	16
67	SCA	8	F	36	8	18	21	2	2	5	2	2	0	3	1	67	33
68	SCA	8	R	23	17	11	40	2	5	1	1	0	0	0	0	38	62
69	SCA	8	R	2	1	27	21	29	18	0	0	0	0	0	0	59	41
70	SCA	8	I	19	5	21	39	3	5	1	3	0	0	3	1	47	53
71	SCA	8	R	7	4	37	31	12	5	3	1	0	0	0	0	59	41
72	SCA	8	F	3	7	32	25	17	13	1	1	0	0	1	0	53	47
73	SCA	8	P	1	2	32	49	5	10	0	0	0	0	0	1	38	62
74	SCA	8	P	0	0	11	49	15	23	0	0	0	0	0	0	27	73
75	SCA	8	B	0	3	35	39	13	9	1	0	0	0	1	0	49	51
76	SCA	8	B	1	3	35	24	25	10	0	0	0	0	0	1	62	38

Table 2a, Table 2b, Table 2ca–c present a summary of the estimated concentrations for potentially bioaccessible or total acid-soluble elements. Elements included in this analysis were Mg, Al, Si, K, V, Cr, Fe, Mn, Co, Ni, Cu, Zn, As, Se, Sr, Ag, Cd, Sn, Ba, Pb, and U.

**Table 2a:** Summary of potentially bioaccessible and total acid-soluble Mg, Al, Si, K, V, Cr, Fe estimated concentrations for 76 respirable coal mine dust samples. Samples are ordered by mine region (i.e., MCA = mid-central Appalachia, NA = northern Appalachia, SCA = south-central Appalachia), mine number (i.e., 1–8), sampling location (i.e., I = intake, R = return, P = production, B = bolter, F = feeder). Elemental concentrations are reported on dry mass basis (i.e., μg per g of respirable dust). Mass values represent dust (μg) recovered from filter. Elemental analysis could not be performed on samples 8 and 30 due to significant dust loss during sample preparation.

Sample					potentially bioaccessible (SLF) and total acid-soluble (total) mass concentration (μg/g)													
					Mg		Al		Si		K		V		Cr		Fe	
No.	Reg.	Mine	Loc.	Mass	SLF	Total	SLF	Total	SLF	Total	SLF	Total	SLF	Total	SLF	Total	SLF	Total
1	MCA	1	F	17	16961	21107	4107	11531	11356	11356	7250	7250	0	0	55	174	1976	32049
2	MCA	1	B	30	14340	27514	9302	54701	15368	21399	11358	11358	0	0	0	191	1143	107093
3	MCA	1	I	2	48598	48598	1576	1576	0	0	14625	14625	0	0	0	12291	320	106817
4	MCA	1	R	43	6622	15862	4001	34026	4469	14402	606	606	0	0	0	0	2621	58879
5	MCA	1	B	5	6993	6993	3204	3204	0	12468	0	0	0	0	0	2400	0	0
6	MCA	1	P	2	61133	82656	13477	44380	0	0	0	0	0	0	0	2701	0	665
7	MCA	1	B	2	303883	702031	206102	706102	423594	423594	184038	184038	0	0	0	2626	44301	544301
8	MCA	2	I	–	–	–	–	–	–	–	–	–	–	–	–	–	–	–
9	MCA	2	R	252	2991	7531	5535	27768	13503	14970	3018	3018	0	0	0	72	4928	41699
10	MCA	2	B	462	2779	9056	2804	33286	8513	10021	113	8449	0	0	0	60	419	54437
11	MCA	2	P	2	77548	100038	24461	168941	55275	55275	115125	115125	0	0	494	11648	22020	347267
12	MCA	2	F	2	69873	69873	27986	70341	3525	3525	143625	143625	0	0	0	4454	42220	219842
13	MCA	2	B	687	2560	12652	3897	35833	8129	15580	4639	14204	0	0	8	97	3709	84081
14	MCA	3	I	2	40848	40848	5244	25724	0	0	226125	226125	0	29566	0	254	0	99622
15	MCA	3	R	9	44516	95597	50875	282926	84617	84617	108639	108639	0	0	0	281	26960	427626
16	MCA	3	B	2	45923	45923	10686	72291	0	0	94375	94375	0	0	0	6216	970	213217
17	MCA	3	P	101	7136	18196	6618	53007	15587	15769	8765	8765	0	0	0	62	643	68599
18	MCA	3	F	5	39419	39419	8554	18297	9010	9010	108850	108850	0	0	0	0	10118	87717
19	MCA	4	I	4	38361	67919	9693	66808	31762	168864	171187	171187	0	0	0	514	10598	99846
20	MCA	4	R	2	10473	10473	0	0	0	0	258875	258875	0	0	0	0	3145	30892
21	MCA	4	B	24	11456	18393	13911	58128	31544	41790	68823	68823	0	0	0	93	8412	122828
22	MCA	4	F	23	5041	5041	1010	1010	589	589	70511	70511	0	0	0	0	1063	3497
23	NA	5	R	101	2605	4003	4592	23886	7656	7656	2922	2922	0	0	0	26	307	15184
24	NA	5	I	2	40533	114931	7124	34277	94	98138	12788	12788	0	0	0	0	0	0
25	NA	5	I	10	12242	16621	3210	13066	0	0	0	0	0	0	0	235	0	0
26	NA	5	F	20	5941	10968	1688	9290	0	442	13004	13004	0	0	0	0	0	0
27	NA	5	P	62	3965	4857	4356	14159	4818	4818	1187	1187	0	0	0	229	0	9699
28	NA	5	R	112	3169	5106	3870	14365	7338	9241	1783	1783	0	0	6	6	0	11151
29	NA	5	B	6	24061	39707	9632	64497	3804	3804	26474	26474	0	0	0	2567	0	171900
30	NA	5	F	–	–	–	–	–	–	–	–	–	–	–	–	–	–	–
31	NA	5	F	175	1230	2018	866	3050	1523	2182	0	0	0	0	0	0	0	4296
32	NA	5	R	2	325883	825883	354371	854371	500000	786685	172171	172171	0	0	0	5276	14405	514405
33	NA	6	I	39	3049	5775	3546	39092	197	197	4897	4897	0	0	0	304	0	36112
34	NA	6	R	2430	177	2363	345	1120	0	212	0	0	0	0	0	4	0	1405
35	NA	6	F	3	26364	36809	29311	151327	43724	43724	16998	16998	0	0	0	0	0	270708
36	NA	6	I	2	27146	29189	6066	11590	23586	23586	0	0	0	0	0	0	0	57562
37	NA	6	R	212	2178	3403	559	1055	798	842	4523	4523	0	0	1606	1606	7013	8463
38	NA	6	P	59	1515	1788	1436	3157	88	3821	0	0	0	0	0	0	236	7281
39	NA	6	I	36	5162	6637	615	1804	0	0	4264	4264	0	0	0	140	0	14941
40	NA	6	R	102	2782	3963	664	1564	2890	3967	3488	3488	0	0	0	116	40	8269
41	NA	6	F	16	5898	7203	1146	4924	302	302	2959	2959	0	1351	0	164	0	8181
42	NA	6	B	11	2829	10136	414	9545	14439	14439	0	0	0	0	0	0	406	13306
43	NA	6	I	29	973	2253	0	989	0	0	2719	2719	0	0	0	0	0	4652
44	NA	6	R	5	3073	3682	1528	1528	0	0	0	0	0	0	0	580	0	0
45	NA	6	I	2	21033	21033	13352	13352	0	0	0	0	0	0	0	1738	0	0
46	NA	6	I	3	48822	48822	10768	16453	0	0	0	0	0	0	0	0	0	0
47	NA	6	F	16	2020	2020	449	449	6918	16127	0	0	0	0	0	0	0	0
48	SCA	7	R	17	7954	16412	8015	42342	187658	201899	0	0	0	0	64	349	2571	35678
49	SCA	7	I	2	16933	23372	0	8344	0	26310	0	0	0	0	0	751	0	9200
50	SCA	7	R	26	11335	31379	14988	101947	33839	45556	19955	19955	0	0	0	346	2760	126948
51	SCA	7	I	2	44033	44033	0	9344	0	30435	0	0	0	2311	0	4476	0	26325
52	SCA	7	F	23	17544	25724	12345	41984	19579	19579	6928	6928	0	0	0	464	1707	48953
53	SCA	7	P	10	26370	35209	5640	30816	9177	41466	10475	10475	0	0	0	50	4265	53127
54	SCA	7	P	24	6475	17075	6240	41751	7032	9194	2594	2594	0	1197	0	58	169	49413
55	SCA	7	F	120	2371	7262	4076	24745	8013	10862	15948	15948	0	0	0	22	531	37040
56	SCA	7	B	90	5375	11701	4921	22724	7324	7324	3722	3722	0	0	0	106	782	22322
57	SCA	7	R	66	6935	19361	10175	58142	17071	17071	6886	6886	0	0	0	102	8258	129010
58	SCA	7	P	39	5073	7902	3307	13616	5863	5863	1961	1961	0	0	0	0	234	11603
59	SCA	7	B	47	5880	24153	10810	79072	20482	20482	10032	10032	0	0	24	24	3488	97416
60	SCA	7	F	72	3367	12989	5585	46638	8086	8086	6875	6875	0	0	0	37	846	67126
61	SCA	7	P	83	2443	6757	3293	24155	8791	8791	4385	4385	0	0	0	122	206	30744
62	SCA	8	R	1048	1849	7732	3095	6917	427	892	288	288	0	0	0	6	98	7071
63	SCA	8	P	22	5731	11584	8725	49930	580	580	11779	11779	0	0	0	168	1114	46204
64	SCA	8	I	12	14198	17158	3462	13692	0	0	761	761	0	0	0	0	0	21769
65	SCA	8	F	46	4625	10951	13255	58766	24745	29393	10068	10068	0	0	0	0	1159	75028
66	SCA	8	I	3	66543	94630	6266	34434	0	66104	12379	12379	0	0	0	0	7001	102329
67	SCA	8	F	3	22159	35997	766	39767	0	0	25046	25046	0	0	0	0	0	67327
68	SCA	8	R	26	12945	25373	16354	91207	28147	28147	21205	21205	0	0	0	0	4489	60187
69	SCA	8	R	143	2590	5755	3557	27580	11178	13626	773	773	0	0	0	6	332	22333
70	SCA	8	I	21	4375	4375	195	2180	563	563	4254	4254	0	553	0	0	101	3036
71	SCA	8	R	19	12309	15815	5818	23288	12438	12438	5413	5413	0	1099	0	0	2866	39322
72	SCA	8	F	53	6136	10232	7611	25286	11418	11418	7670	7670	0	0	0	219	5840	34568
73	SCA	8	P	189	2628	9865	3944	38482	10868	12763	3188	3188	0	0	0	0	390	50078
74	SCA	8	P	1626	1304	2996	3458	12929	6293	7533	1348	3972	0	0	9	18	4158	16346
75	SCA	8	B	82	5055	10230	6336	40736	11441	11441	6872	6872	0	0	0	45	261	29738
76	SCA	8	B	112	1598	3970	3467	24742	8628	11011	2328	2328	0	0	0	0	204	9176

**Table 2b:** Summary of potentially bioaccessible and total acid-soluble Mn, Co, Ni, Cu, Zn, As, Se estimated concentrations for 76 respirable coal mine dust samples. Samples are ordered by mine region (i.e., MCA = mid-central Appalachia, NA = northern Appalachia, SCA = south-central Appalachia), mine number (i.e., 1–8), sampling location (i.e., I = intake, R = return, P = production, B = bolter, F = feeder). Elemental concentrations are reported on dry mass basis (i.e., μg per g of respirable dust). Mass values represent dust (μg) recovered from filter. Elemental analysis could not be performed on samples 8 and 30 due to significant dust loss during sample preparation.

Sample					potentially bioaccessible (SLF) and total acid-soluble (total) mass concentration (μg/g)													
					Mn		Co		Ni		Cu		Zn		As		Se	
No.	Reg.	Mine	Loc.	Mass	SLF	Total	SLF	Total	SLF	Total	SLF	Total	SLF	Total	SLF	Total	SLF	Total
1	MCA	1	F	17	84	84	3	3	172	172	719	719	2545	2545	0	0	0	0
2	MCA	1	B	30	243	2158	10	10	234	1366	277	776	0	482052	0	0	0	0
3	MCA	1	I	2	0	0	0	0	704	5065	0	0	0	0	0	0	0	0
4	MCA	1	R	43	396	1274	1	1	26	45	0	0	0	0	0	0	0	0
5	MCA	1	B	5	0	0	18	18	670	1054	61	61	2367	2367	0	0	0	0
6	MCA	1	P	2	1364	1364	11	11	728	728	0	0	0	0	0	0	0	0
7	MCA	1	B	2	9904	54074	104	104	460	460	1226	1226	0	0	0	0	0	0
8	MCA	2	I	–	–	–	–	–	–	–	–	–	–	–	–	–	–	–
9	MCA	2	R	252	313	953	5	18	27	45	0	0	0	0	0	0	0	0
10	MCA	2	B	462	339	1316	4	24	0	24	0	0	0	389	0	7	0	0
11	MCA	2	P	2	1398	1398	67	67	2309	2309	970	970	0	0	0	0	0	0
12	MCA	2	F	2	1901	1901	114	114	1109	1109	0	0	6411	6411	0	0	0	0
13	MCA	2	B	687	352	1914	3	32	4	71	0	82	0	131	0	0	0	0
14	MCA	3	I	2	0	0	0	0	1084	1320	0	0	1861	1861	0	4704	0	0
15	MCA	3	R	9	3420	8646	89	89	342	342	907	907	269	269	0	0	0	0
16	MCA	3	B	2	1113	1113	4	4	539	539	0	0	0	0	0	0	0	0
17	MCA	3	P	101	332	1291	7	43	7	105	0	0	0	0	0	0	0	0
18	MCA	3	F	5	340	340	0	0	277	277	745	745	4894	4894	0	0	0	0
19	MCA	4	I	4	688	688	37	37	672	1221	138	138	1818	1818	0	0	0	0
20	MCA	4	R	2	0	0	0	0	694	694	318	318	0	0	0	0	0	0
21	MCA	4	B	24	818	2947	26	26	64	94	121	121	0	0	0	339	0	0
22	MCA	4	F	23	17	17	8	8	86	86	741	741	1147	1147	0	0	0	0
23	NA	5	R	101	127	127	8	24	9	33	0	0	0	0	0	0	0	0
24	NA	5	I	2	776	776	9	9	445	445	4963	4963	10291	10291	0	0	0	0
25	NA	5	I	10	180	180	0	0	88	562	34	34	0	0	0	0	0	0
26	NA	5	F	20	84	84	0	0	10	7042	0	0	0	0	0	0	0	0
27	NA	5	P	62	43	43	7	7	7	7	52	52	0	0	1	1	0	0
28	NA	5	R	112	43	43	4	4	7	7	0	0	0	0	0	0	0	0
29	NA	5	B	6	752	752	21	21	149	612	220	220	0	0	0	0	0	0
30	NA	5	F	–	–	–	–	–	–	–	–	–	–	–	–	–	–	–
31	NA	5	F	175	16	16	0	0	4	4	0	0	0	0	0	0	0	0
32	NA	5	R	2	1744	20709	107	1187	432	5633	1889	1889	0	500000	0	0	0	0
33	NA	6	I	39	273	273	4	4	52	196	0	896	0	134545	0	0	0	0
34	NA	6	R	2430	0	53	0	4	0	10	0	0	0	0	0	2	0	0
35	NA	6	F	3	2537	2537	33	33	285	1209	0	0	0	101250	0	7	0	0
36	NA	6	I	2	0	0	54	54	651	1285	0	0	0	169124	0	810	0	0
37	NA	6	R	212	177	177	11	11	730	730	0	0	89	89	0	0	0	0
38	NA	6	P	59	47	47	7	7	19	109	0	0	0	102538	0	0	0	0
39	NA	6	I	36	0	0	7	7	32	216	0	0	0	0	0	0	0	0
40	NA	6	R	102	18	18	2	2	17	17	26	26	258	258	0	0	0	0
41	NA	6	F	16	101	101	25	25	115	115	54	54	0	300409	0	151	0	0
42	NA	6	B	11	171	171	0	0	85	110	0	0	984	18806	0	0	0	0
43	NA	6	I	29	0	0	0	0	95	535	0	0	0	92639	0	0	0	0
44	NA	6	R	5	0	0	0	0	49	49	0	0	0	0	0	216	0	0
45	NA	6	I	2	0	0	0	0	150	150	0	0	0	0	0	0	0	0
46	NA	6	I	3	398	398	3	3	0	0	0	0	0	0	0	0	0	0
47	NA	6	F	16	0	0	0	0	23	100	0	0	3611	3611	0	0	0	0
48	SCA	7	R	17	236	236	5	5	78	78	108	108	0	0	0	0	0	0
49	SCA	7	I	2	0	0	37	37	40	53	0	0	0	0	0	0	0	0
50	SCA	7	R	26	734	2469	16	16	27	142	326	881	0	62078	0	0	0	0
51	SCA	7	I	2	0	0	7	7	625	950	3029	3029	0	500000	0	0	0	0
52	SCA	7	F	23	198	198	0	0	7	172	54	54	0	45719	0	0	0	0
53	SCA	7	P	10	494	494	15	15	448	448	0	0	0	0	0	0	0	0
54	SCA	7	P	24	241	241	10	10	60	60	0	0	0	0	0	0	0	0
55	SCA	7	F	120	155	606	2	13	19	33	33	33	250	250	0	0	0	0
56	SCA	7	B	90	55	298	6	6	10	19	0	0	196	4488	0	0	0	0
57	SCA	7	R	66	595	2021	8	43	18	18	0	0	0	3076	0	0	0	0
58	SCA	7	P	39	61	61	5	5	25	56	0	0	0	0	0	0	0	0
59	SCA	7	B	47	234	1492	7	59	18	23	0	0	0	0	0	0	0	0
60	SCA	7	F	72	230	1201	5	30	16	25	0	0	0	1219	0	0	0	0
61	SCA	7	P	83	176	531	3	21	9	30	0	0	0	3370	0	0	0	0
62	SCA	8	R	1048	13	77	0	3	2	11	0	0	0	940	0	0	0	0
63	SCA	8	P	22	276	276	4	4	59	59	34	34	0	30784	0	0	0	0
64	SCA	8	I	12	122	122	0	0	96	439	0	0	2259	2259	0	0	0	0
65	SCA	8	F	46	141	666	0	28	22	67	1	1	0	0	0	0	0	0
66	SCA	8	I	3	453	453	49	49	352	352	1040	1040	0	0	0	0	0	0
67	SCA	8	F	3	440	440	0	0	290	290	0	0	5620	5620	0	982	0	0
68	SCA	8	R	26	194	194	6	6	40	57	74	74	1063	17728	0	0	0	0
69	SCA	8	R	143	162	458	4	15	3	3	0	0	0	8755	0	0	0	0
70	SCA	8	I	21	0	0	1	1	26	26	139	139	0	0	0	0	0	0
71	SCA	8	R	19	247	247	12	12	17	67	226	226	0	15857	0	0	0	0
72	SCA	8	F	53	176	176	7	7	12	12	40	40	0	0	0	1	0	0
73	SCA	8	P	189	141	869	0	17	5	25	0	0	0	0	0	0	0	0
74	SCA	8	P	1626	108	288	3	10	3	13	0	0	0	0	0	0	0	0
75	SCA	8	B	82	107	493	1	20	5	87	21	21	0	6091	0	0	0	0
76	SCA	8	B	112	36	36	3	3	6	6	1	1	0	0	0	0	0	0

**Table 2c:** Summary of potentially bioaccessible and total acid-soluble Sr, Ag, Cd, Sn, Ba, Pb, U estimated concentrations for 76 respirable coal mine dust samples. Samples are ordered by mine region (i.e., MCA = mid-central Appalachia, NA = northern Appalachia, SCA = south-central Appalachia), mine number (i.e., 1–8), sampling location (i.e., I = intake, R = return, P = production, B = bolter, F = feeder). Elemental concentrations are reported on dry mass basis (i.e., μg per g of respirable dust). Mass values represent dust (μg) recovered from filter. Elemental analysis could not be performed on samples 8 and 30 due to significant dust loss during sample preparation.

Sample					potentially bioaccessible (SLF) and total acid-soluble (total) mass concentration (μg/g)																
					Sr		Ag			Cd			Sn			Ba		Pb		U	
No.	Reg.	Mine	Loc.	Mass	SLF	Total		SLF	Total		SLF	Total		SLF	Total	SLF	Total	SLF	Total	SLF	Total
1	MCA	1	F	17	0	0		0	411		0	0		0	0	38	38	0	0	0	0
2	MCA	1	B	30	0	0		0	159		0	0		0	0	90	90	0	0	0	0
3	MCA	1	I	2	0	0		332	999		0	0		0	0	6519	6519	0	0	0	0
4	MCA	1	R	43	0	0		0	102		0	0		0	0	374	374	0	0	0	0
5	MCA	1	B	5	0	0		0	0		0	0		0	0	3240	3240	0	0	0	0
6	MCA	1	P	2	0	0		0	387		0	0		0	0	4475	4475	0	0	0	0
7	MCA	1	B	2	0	0		0	1612		0	0		0	0	3525	3525	0	0	0	0
8	MCA	2	I	–	–	–		–	–		–	–		–	–	–	–	–	–	–	–
9	MCA	2	R	252	0	0		0	105		0	0		0	0	92	92	0	0	0	0
10	MCA	2	B	462	0	0		0	233		0	0		0	0	12	12	0	76	0	3
11	MCA	2	P	2	0	0		0	0		0	0		0	0	2469	2469	0	0	0	0
12	MCA	2	F	2	0	0		0	291		0	0		0	0	0	0	0	0	0	0
13	MCA	2	B	687	0	0		0	29		0	0		0	0	194	194	0	75	0	2
14	MCA	3	I	2	0	0		0	1266		0	0		0	0	0	0	0	0	0	0
15	MCA	3	R	9	0	0		0	884		0	0		0	0	0	0	0	0	0	0
16	MCA	3	B	2	0	0		0	204		0	0		0	0	0	0	0	0	0	0
17	MCA	3	P	101	0	0		0	111		0	0		0	0	22	22	0	0	0	0
18	MCA	3	F	5	0	0		0	11		0	0		0	0	0	0	0	0	0	0
19	MCA	4	I	4	0	0		0	0		0	0		0	0	0	0	0	5769	0	0
20	MCA	4	R	2	0	0		0	0		0	0		0	0	0	0	0	0	0	0
21	MCA	4	B	24	0	0		0	272		0	0		0	0	0	0	0	0	0	0
22	MCA	4	F	23	0	0		0	11		0	0		0	0	0	0	0	0	0	0
23	NA	5	R	101	0	0		0	25		0	0		0	0	0	0	0	0	0	0
24	NA	5	I	2	0	0		0	1174		0	0		0	0	4275	4275	0	0	0	0
25	NA	5	I	10	0	0		0	180		0	0		0	0	0	0	0	0	0	0
26	NA	5	F	20	0	0		0	76		0	0		0	0	143	143	0	0	0	0
27	NA	5	P	62	0	0		0	134		0	0		0	0	0	0	0	0	0	0
28	NA	5	R	112	0	0		0	66		0	0		0	0	0	0	0	0	0	0
29	NA	5	B	6	0	0		0	0		0	0		0	0	0	0	0	0	0	0
30	NA	5	F	–	–	–		–	–		–	–		–	–	–	–	–	–	–	–
31	NA	5	F	175	0	0		20	47		0	0		0	0	0	0	0	0	0	0
32	NA	5	R	2	41962	41962		0	0		0	0		0	0	0	0	0	0	0	0
33	NA	6	I	39	0	0		0	60		0	0		0	0	0	0	0	0	0	0
34	NA	6	R	2430	0	276		3	29		0	0		0	0	0	0	0	18	0	0
35	NA	6	F	3	0	0		0	6716		0	0		0	0	0	0	0	0	0	0
36	NA	6	I	2	0	0		0	0		0	0		0	0	0	0	0	0	0	0
37	NA	6	R	212	0	0		5	27		0	0		0	0	0	0	13	13	0	0
38	NA	6	P	59	0	0		0	0		0	0		0	0	0	0	0	0	0	0
39	NA	6	I	36	0	0		0	0		0	0		0	0	0	0	0	0	0	0
40	NA	6	R	102	0	0		31	36		0	0		0	0	301	301	0	0	0	0
41	NA	6	F	16	0	0		0	0		0	0		0	0	0	0	0	0	0	0
42	NA	6	B	11	0	0		0	0		0	0		0	0	0	0	0	0	0	0
43	NA	6	I	29	0	0		0	0		0	0		0	0	0	0	0	0	0	0
44	NA	6	R	5	0	0		0	0		0	0		0	0	0	0	0	0	0	0
45	NA	6	I	2	0	0		0	974		0	0		0	0	0	0	0	0	0	0
46	NA	6	I	3	0	0		0	0		0	0		0	0	0	0	0	0	0	0
47	NA	6	F	16	0	0		0	1792		0	0		0	0	0	0	0	0	0	0
48	SCA	7	R	17	0	0		0	10		0	0		0	0	0	0	0	0	0	0
49	SCA	7	I	2	0	0		0	0		0	0		0	0	0	0	0	0	0	0
50	SCA	7	R	26	0	0		0	0		0	0		0	0	0	0	0	0	0	0
51	SCA	7	I	2	0	0		0	0		0	0		0	0	0	0	0	0	0	0
52	SCA	7	F	23	0	0		0	0		0	0		0	0	0	0	0	0	0	0
53	SCA	7	P	10	0	0		0	408		0	0		0	0	0	0	0	0	0	0
54	SCA	7	P	24	0	0		0	206		0	0		0	0	0	0	0	0	0	0
55	SCA	7	F	120	0	0		0	26		0	0		0	0	27	27	0	525	0	0
56	SCA	7	B	90	0	0		0	40		0	0		0	0	0	0	0	0	0	0
57	SCA	7	R	66	0	0		0	45		0	0		0	0	0	0	0	0	0	0
58	SCA	7	P	39	0	0		0	73		0	0		0	0	0	0	0	0	0	0
59	SCA	7	B	47	0	0		0	79		0	0		0	0	0	0	0	0	0	0
60	SCA	7	F	72	0	0		0	27		0	0		0	0	69	69	0	0	0	0
61	SCA	7	P	83	0	0		0	27		0	0		0	0	0	0	0	0	0	0
62	SCA	8	R	1048	0	0		0	36		0	0		0	0	0	0	0	50	0	0
63	SCA	8	P	22	0	0		0	264		0	0		0	0	0	0	0	0	0	0
64	SCA	8	I	12	0	0		0	2738		0	0		0	0	6	6	0	0	0	0
65	SCA	8	F	46	0	0		0	88		0	0		0	0	273	273	0	0	0	0
66	SCA	8	I	3	0	0		356	667		0	0		0	0	0	0	0	0	0	0
67	SCA	8	F	3	0	0		0	0		0	0		0	0	0	0	0	0	0	0
68	SCA	8	R	26	0	0		0	2101		0	0		0	0	384	384	0	0	0	0
69	SCA	8	R	143	0	0		0	46		0	0		0	0	0	0	0	0	0	0
70	SCA	8	I	21	0	0		0	0		0	0		0	0	0	0	0	0	0	0
71	SCA	8	R	19	4088	4088		0	0		0	0		0	0	0	0	0	0	0	0
72	SCA	8	F	53	0	0		0	32		0	0		0	0	123	123	0	0	0	0
73	SCA	8	P	189	0	0		0	53		0	0		0	0	172	172	0	0	0	0
74	SCA	8	P	1626	0	0		0	16		0	0		0	0	7	7	0	28	0	0
75	SCA	8	B	82	0	0		0	36		0	0		0	0	67	67	0	0	0	0
76	SCA	8	B	112	0	0		0	16		0	0		0	0	12	12	0	0	0	0

## 2 Experimental design, materials, and methods

### 2.1 Sample collection

A total of 76 sets of respirable dust samples were collected in eight underground coal mines in mid-central (MCA, mines 1–4), northern (NA, mines 5 and 6), and south-central Appalachia (SCA, mines 7 and 8). The samples were collected in five key locations: intake airway (I), just outby of the primary production area (including the headgate of a longwall section) or along the mantrip track; feeder (F), near the feeder breaker or along the main conveyor belt; production (P), just downwind of an active continuous miner or near the midface of a longwall section (except for Mine 4); roof bolter (B), just downwind of an active bolter; and return airway (R), just outby of the primary production area (including the tailgate of a longwall section).

A detailed description of the mines and sampling protocol was previously reported [2]. Briefly, all samples were collected using a small air pump with a 10-mm nylon Dorr-Oliver cyclone, which produces a d_50_ cut size of about 4 μm at the sampling flow rate of 1.7 L/min. Each sample set represents a unique sampling event, during which multiple replicate samples were collected simultaneously, over a continuous 2–4 hr period. One sample from each set is included in the analysis described here. These samples were collected directly onto 37-mm polycarbonate filters (PC, track-etched with 0.4 μm pore size).

Although the PC filters used in this study are expected to have very high overall collection efficiencies for the particle size range studied here (e.g., see [3], [4]), at least some penetration of very fine particles likely occurred. Surface collection efficiencies were probably impacted more. For example, others have previously observed that PC filters (0.29 μm pore size, 1L/min sample flow rate) had surface collection efficiencies of 22, 42 and 83% for 75, 133, and 237nm particles, respectively [4]. Results reported in Table 1 and Fig. 1 should be viewed accordingly.

As described in the companion research article [1], a 9-mm circular subsection was cut from the center of each PC filter sample and prepared for particle distribution analysis by sputter-coating with Au/Pd; and the rest of the filter was used for the metals and trace elements analysis.

### 2.2 Particle distribution analysis

The particle distribution analysis was conducted in two phases: supramicron and submicron. The supramicron analysis was performed using a computer-controlled SEM-EDX routine, which has been described elsewhere [5], and the results were previously reported in another research article [2]. The submicron analysis was performed using manual SEM-EDX, which is described in detail below. Both phases of analysis were done using the same instrumentation and software, a FEI Quanta 600 FEG environmental SEM (FEI, Hillsboro, OR) equipped with a Bruker Quantax 400 EDX spectroscope (operated in backscatter mode) and Esprit software (Version 1.9) (Bruker, Ewing, NJ). Table 3 highlights key features of each analytical routine.

**Table 3:** Description of sub- and supramicron particle analysis routines using SEM-EDX.

Feature	Submicron Analysis	Supramicron Analysis
Method	Manual	Computer Controlled
Magnification	20,000x	1,000x
Spot size	4	6.5
Voltage (kV)	10	15
Working distance (mm)	12.5	12.5
# Frames/sample, range	17–189	10–157
# Frames/sample, average	69	33
# Particles/frame, max	7	50
# Particles/sample, range	83–315	61–500
# Particles/sample, average	236	489
Diameter (nm)	∼100–1000	∼1000–10,000
Classification by	Elemental spectral peak heights (Cps/eV)	Atomic % determined from elemental spectral peak ratios (only considering C, O, Al, Si, Ca, Mg, Fe, Ti)
Typical particle types	C = diesel particulates, coal dust	C = coal dust
	AS = clay mineral dust	AS = clay mineral dust
	S = silica dust	S = silica dust
	CB = carbonate mineral dust	CB = carbonate mineral dust
	HM = not often possible to identify	HM = metal sulfide/oxide dust

In both phases of analysis, individual particles were selected for analysis. While filter overloading was not generally an issue, care was taken to only select non-aggregated particles (i.e., distinct from neighboring particles). This approach served to minimize interference between particles for elemental analysis, but does assume that the distribution of non-aggregated particles on the sample filters is representative of the overall particle distribution. For each particle, two main types of data were collected: (1) dimensions, and (2) the elemental spectra. The dimensions were used to determine particle projected area diameters. The spectral peak heights (or their resulting atomic ratios) were used to classify particles by their mineralogy. Classification criteria for supramicron particles into five defined mineralogy classes (i.e., C = carbonaceous, AS = alumino-silicates, S = silica, CB = carbonates, HM = heavy minerals) were previously described [5]. Those criteria were developed and verified using particles from high-purity or known materials. Any particles that did not meet the criteria for one of the five defined classes was binned into a class called “other” (i.e., O).

**Fig. 2:**
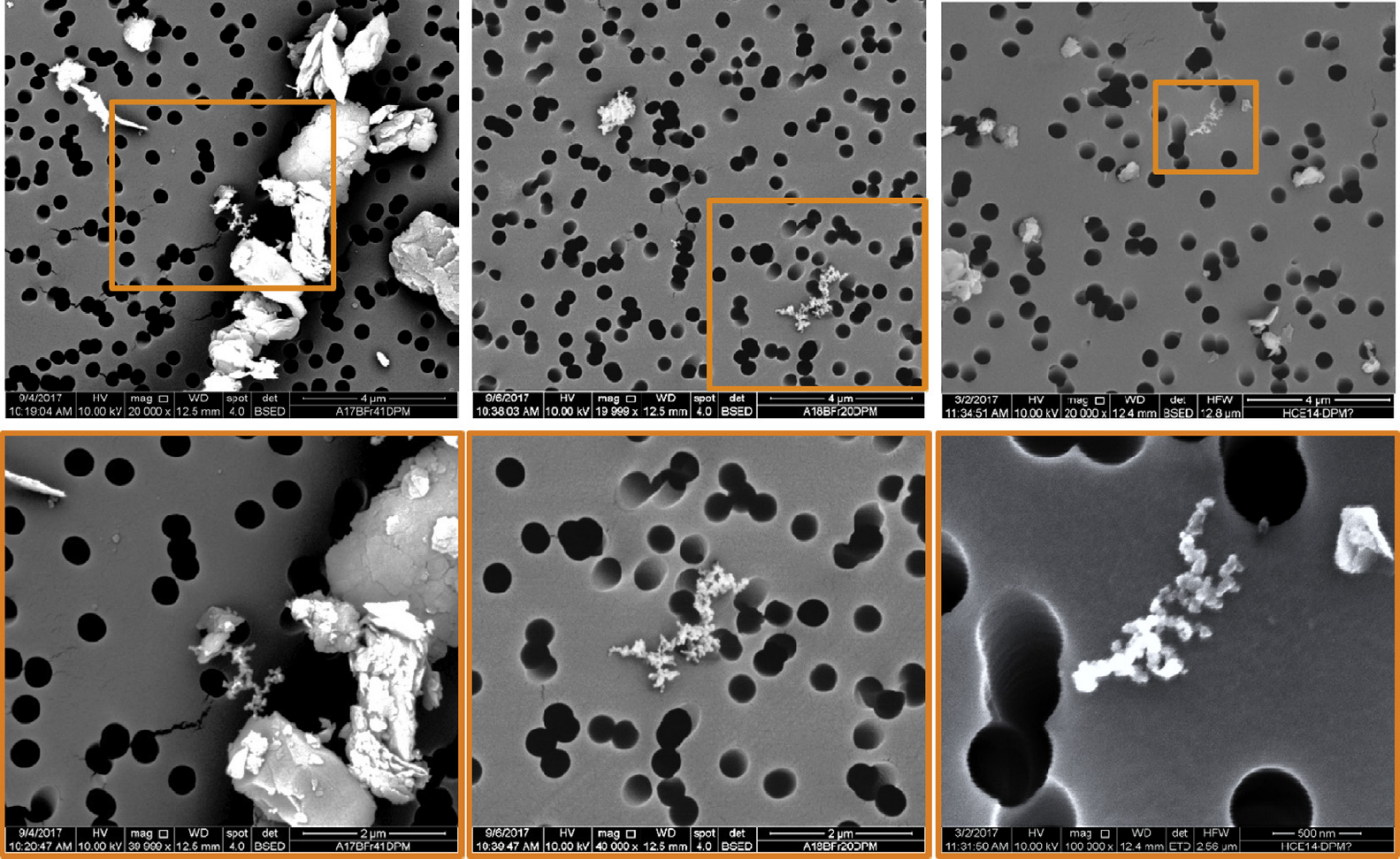
Examples of DPM particles in dust samples from several mines. In some cases, DPM can be identified by its characteristic morphology of chains or clusters of ultrafine carbon spheres. Images in the upper panel were collected at the 20,000x standard magnification used for the manual SEM-EDX work to characterize submicron particles. Images in the lower panel were collected at 30,000x (left), 40,000x (middle) and 100,000x (right).

The classification criteria were adapted (Table 4) and verified (Table 5) for analysis of submicron particles, using a similar approach. It should be noted that submicron particles in the C class may include both carbonaceous (i.e., coal) dust and diesel particulates, which can sometimes be identified based on their characteristic morphology (e.g., as shown in Fig. 2).

**Table 4:** Classification criteria for each defined mineralogy category used in the sub- and supramicron particle analysis. (Supramicron criteria were previously published [5]). The values represent minimum raw spectral peak heights (Cps/eV) for the manual submicron analysis and minimum atomic percentage for the automated supramicron analysis under the SEM-EDX instrument settings noted in Table 3. Reproduced from the companion research article [1].

Element	Submicron (Cps/eV)					Supramicron (Atomic %)				
	C	AS	S	CB	HM	C	AS	S	CB	HM
Carbon	≥48	–	–	–	–	>74	<85	<86	<85	–
Oxygen	Not included					<29	>13	>15	>15	>12
Aluminum	–	≥0.5	–	–	–	<0.3	>0.2	<0.2	–	–
Silicon	–	≥0.5	≥0.2	–	<0.5	<0.3	>0.2	>0.5	–	–
Calcium/Magnesium	–	–	–	≥0.3	–	<0.3	–	–	>0.5	–
Iron/Titanium/Aluminum	–	–	–	–	≥0.5	–	–	–	–	>0.5

**Table 5:** Classification results on submicron particles in respirable dust samples generated in the laboratory using high-purity or known materials. Results are shown for particles both above and below the 400nm threshold used to delineate very fine and larger particles in this work. The coal material was known to have some mineral content associated with it; analysis on a −325 mesh (i.e., −44 μm) bulk sample of the material showed about 10% ash by mass, and mineral content is expected to concentrate in finer size fractions. Reproduced from the companion research article [1].

Dust Source Material	Classification Category											
	C		AS		S		CB		HM		Other	
	<400	≥400	<400	≥400	<400	≥400	<400	≥400	<400	≥400	<400	≥400
Coal	74%	63%	26%	37%	0%	0%	0%	0%	0%	0%	0%	0%
Shale	3%	0%	88%	88%	9%	13%	0%	0%	0%	0%	1%	0%
Rock Dust	1%	0%	15%	14%	0%	0%	84%	84%	0%	0%	0%	2%
Quartz	0%	0%	6%	8%	93%	90%	0%	0%	0%	1%	1%	0%
Kaolinite	9%	0%	90%	89%	0%	0%	0%	5%	1%	5%	0%	0%
Calcite	3%	3%	2%	0%	0%	0%	92%	97%	2%	0%	2%	0%

**Fig. 3:**
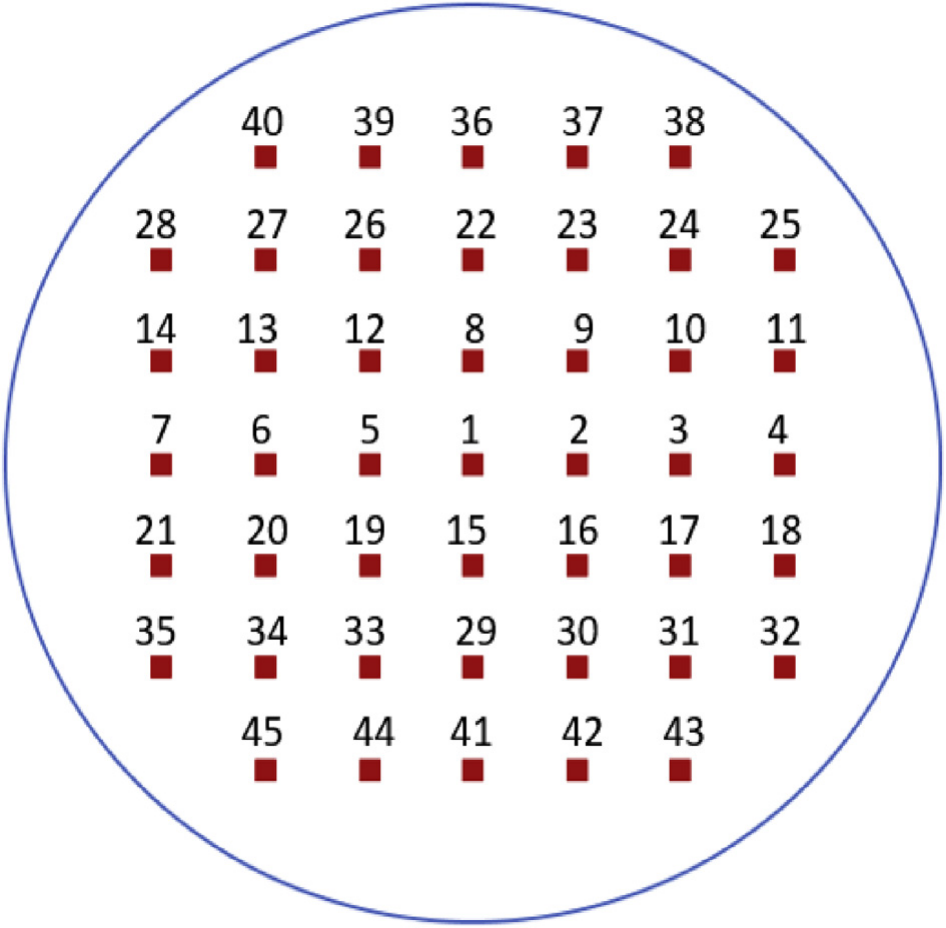
Frame positions for manual SEM-EDX analysis of submicron particles on a 9-mm subsection of a filter sample.
• In each frame, seven particles were analyzed. The first four were in the upper left quadrant of the frame; and the last three were in the lower right quadrant (see Fig. 4). This means that the maximum number of particles selected for analysis was 315 (i.e., 7 particles per frame by 45 frames). For each particle, the long and intermediate dimensions were measured, and then EDX elemental spectral peak heights (cps/eV) were recorded for the following elements: C, Al, Si, Ca, Mg, Fe, Ti, S, K, Na, P, Cr, Ni, Cl, Mn, Cu, Zn, Pb, Hf, Co, and F. Using these peak heights, the particle could be binned into one of the five defined mineralogy classes (i.e., C, AS, S, CB, HM per Table 4) or into the O class.

The manual submicron particle analysis proceeded as follows:

• Initially, the SEM stage was moved to the center frame (i.e., in the center of the sample). Analysis began at the center frame and then proceeded through subsequent frames respectively (from frame 1 to 45, see Fig. 3) to ensure particle selection across a wide area. At 20,000x magnification, each frame was approximately 139 μm^2^ (12.67 μm × 10.96 μm) and the frames were spaced 1 mm apart.

**Fig. 4:**
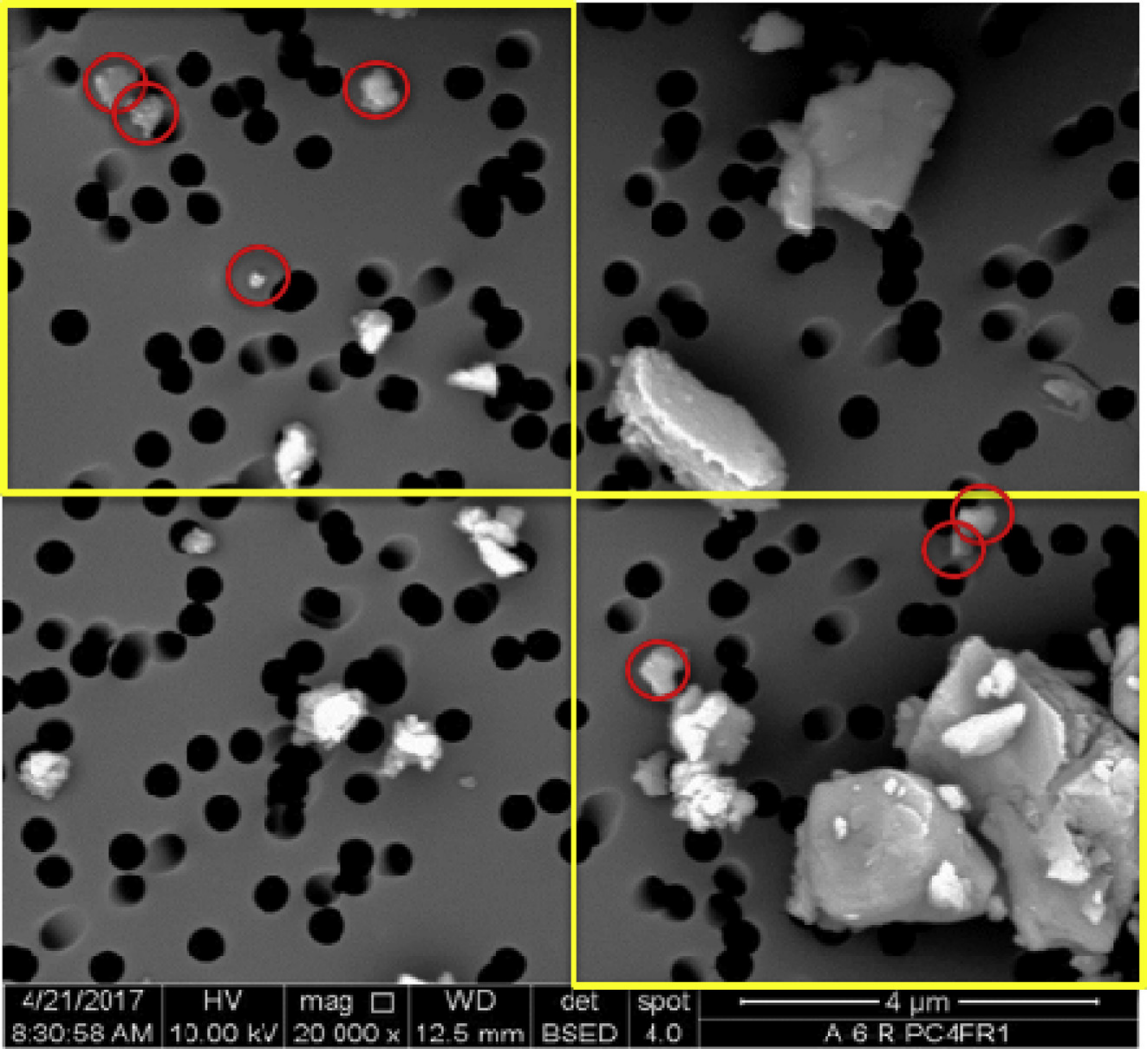
Illustrative example of analysis in upper left and lower right quadrants of an SEM-EDX frame for submicron particle analysis. In this particular frame, the seven circled particles would be selected for analysis.
• If particle loading on the sample was relatively light, the upper right and lower left quadrants in each frame were used to identify additional particles (i.e., up to the maximum of 7 per frame). Further, more frames were analyzed beyond the 45 shown in Fig. 3 if necessary; in this case, frames were located equidistant between the those identified in Fig. 3 and analysis proceeded following the same pattern.

Following completion of both the sub- and supramicron particle analysis, the resulting datasets were merged to allow description of particle distributions across the entire size range (Fig. 1). This was done by normalizing both datasets on the basis of particles per analyzed-filter-area. Finally, the data were split into the very fine (i.e., <400nm) and larger particles (i.e., 400–10,000nm) bins included in Table 1.

### 2.3 Metals and trace elements analysis

Following is a detailed description of the method used to prepare and analyze respirable coal mine dust samples to determine mass concentrations of potentially bioaccessible and total acid-soluble metals and trace elements. The method involved two sequential digestions of each dust sample, the first in a simulated lung fluid (SLF) and the second in a strong acid solution. It is noted that the term “total acid-soluble” used here refers to the total mass concentration of each element that could be dissolved via both digestions. Because the strong acid digestion did not employ hydrofluoric acid (HF), it is also noted that some elemental concentrations (i.e., particularly Si and Al) could be underestimated.

First, dust was recovered from each PC filter remnant (i.e., following removal of the 9-mm subsection used for SEM-EDX work):

• Each filter remnant was weighed to establish a pre-weight prior to dust removal.
• Each filter was then placed into a glass digestion tube and rinsed with 18 MΩ water. Enough water was added to fully submerse the filter. The tubes were then capped and sonicated for 1 hr, followed by centrifuging for 10 min (@ 3000 rpm) to settle the dust. Tubes were then uncapped and water was evaporated in a clean oven (@110 °C).
• Dry filters were re-weighed to determine recovered dust mass. For the purpose of estimating elemental concentrations in the current study (Table 2a, Table 2b, Table 2ca–c), any dust mass measured as <2 μg was assumed to be 2 μg to limit calculation of inordinately high concentrations.
Next, SLF, which is sometimes called “Gamble's solution”, was prepared per [6]:
• To prepare 1 L of SLF, the ingredients shown in Table 6 were added (in listed order) to 1 L of 18 MΩ water, which was gently mixing by magnetic stirrer.

**Table 6:** SLF solution chemistry. All chemicals were reagent-grade or higher where available.

Addition	Chemical	Formula	Concentration/L
1	Ammonium chloride	NH_4_Cl	535 mg
2	Sodium chloride	NaCl	6780 mg
3	Sodium bicarbonate	NaHCO_3_	1770 mg
4	Sodium carbonate	Na_2_CO_3_	630 mg
5	Sodium dihydrogen phosphate monohydrate	NaH_2_PO_4_·H_2_O	166 mg
6	Sodium citrate dihydrate	Na_3_-citrate·2H_2_O	59 mg
7	Glycine	C_2_H_5_NO_2_	450 mg
8	Sulfuric acid	H_2_SO_4_	51 mg (27.7 μL)
9	Calcium chloride dihydrate	CaCl_2_·2H_2_O	29 mg
• The solution was then placed in a water bath (constant 37 °C), and the pH was adjusted to 7.4 using trace-metal grade HCl.
• The SLF solution was added to each digestion tube containing dry dust, as well as tubes prepared as matrix and blank samples. The SLF solution volume was determined using a 1/50,000 solid (i.e., dust) to SLF liquid ratio per [7]. They recommend a ratio between 1/500 and 1/50,000 for experiments to estimate bioaccessibility of metals. Since the dust samples available for this study generally had low weights (i.e., below 1 mg), and at least 5 mL of solution is required for the ICP-MS elemental analysis, the maximum recommended solid to SLF liquid ratio was adopted.
• The tubes were capped and placed in the sonication bath for 24 hours (@ constant 37 °C), and then centrifuged for 10 min (@ 3000 rpm).
• A 5 mL aliquot of the liquid was taken by syringe using a PTFE filter (0.1 μm pore size), to trap any remaining dust particles, and then the SLF digestate was added to an ICP tube and acidified to 2% (by volume) HNO3 using trace-metal grade acid.
Then, a method modified from ASTM D7439-14 [8] was used to digest the remaining dust from each sample (i.e., that not digested by the SLF) in a strong acid solution:
• The PTFE filter used to trap dust from the SLF sample was placed back into the tube used for the SLF digestion. The filter and tube walls were then washed by pipetting a solution of 10% HNO_3_ (prepared with 18 MΩ water).
• Under a fume hood, the tubes were positioned in a hot block (internal temperature @ 95 °C), covered with watch glass, and heated until completely dry.
• Then 1.25 mL of concentrated HCl was added to each tube, and the tubes were again covered and placed back into the hot block for 15 min, followed by 5 min of cooling.
• The above step was then repeated with 1.25 mL of concentrated HNO_3_.
• Each tube was then diluted to a final volume of 25 mL with 18 MΩ water, taking care to wash down the sides of the tube and watch glass, and capped and shaken.
• A 5 mL aliquot of the liquid was taken by syringe using a PTFE filter (0.1 μm pore size), to trap any remaining dust particles, and then the strong acid digestate was added to an ICP tube and acidified to 2% (by volume) HNO_3_ using trace-metal grade acid.

Finally, digestates from the SLF and strong acid digestions were analyzed by ICP-MS using a Thermo Electron X Series instrument (Thermo Fisher Scientific, Waltham, MA):

• For each ICP run, at least 5 blank PC filters were prepared using both SLF and strong acid digestion procedures to allow for blank corrections. The SLF and strong acid solutions were also analyzed to allow matrix corrections.
• ICP results (μg/L in the digestate solutions) were corrected and then transformed into dry dust concentrations (μg/g) using the dust mass recovered from each filter. The concentration determined from the SLF digestate is regarded as potentially bioaccessible; and the sum of the concentration from the SLF and strong acid digestates is regarded as total acid-soluble concentration. It is noted that, due to relatively low sample masses for the current dataset, results in Table 2a, Table 2b, Table 2ca–c should be regarded as estimated concentrations.
• The elements that were measured by ICP-MS and reported here are listed in Table 7 with their respective method reporting level (MRL) in the ICP solution. These limits are based on the calibration curve for each element, which is generated using a series of standard solutions. The limits of detection are generally about one order of magnitude lower. (Note that other elements, including Ca, Na, P, Ti, S and Cl, can be measured by ICP-MS, but were not included in the analysis presented here due to significant interferences from the digestion solutions.) In addition to ICP-MS calibration prior to sample analysis, check standards and blank samples were run between every set of 10 samples analyzed to ensure that there was no significant instrument drift or carryover contamination between samples.

**Table 7:** MRLs for elements included in ICP-MS analysis.

MRL (μg/L)	Element	MRL (μg/L)	Element
0.05	U	5	Se
0.1	Co, Ni, Ag	10	Fe, Mg, Si, Sr, V, Zn
0.5	As	50	Ba
1	Al, Cd, Cr, Cu, Pb, Mn	100	K
