# Supplementary material for: Characteristics of respirable dust in eight appalachian coal mines: A dataset including particle size and mineralogy distributions, and metal and trace element mass concentrations
Source: Data Brief. 2019 May 22;25:104032. doi: 10.1016/j.dib.2019.104032 (PMC6557733; doi:10.1016/j.dib.2019.104032)
Supplement: Multimedia component 1 [file mmc1.pdf]

**CONFLICT OF INTEREST**

***Author information:***

**Name and Surname:** Emily Sarver

**Address:** 108A Holden Hall, Old Turner Street, Virginia Tech, Blacksburg, VA 24061 USA

**Email:** esarver@vt.edu

***Article information:***

**Title:** Characteristics of respirable dust in eight Appalachian coal mines: a dataset including particle size and mineralogy distributions, and metal and trace element mass concentrations

***Conflict of Interest:***

**Author states that there is not any conflict of interest**

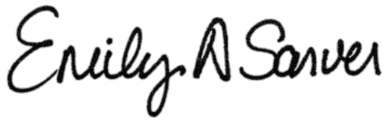

1 May 2019, Blacksburg, VA

**Date and place of signature**
